# Synthesis and Inhibitory Effect of Some Indole‐Pyrimidine Based Hybrid Heterocycles on α‐Glucosidase and α‐Amylase as Potential Hypoglycemic Agents

**DOI:** 10.1002/open.201900240

**Published:** 2019-10-22

**Authors:** Farid A. Badria, Saleh Atef, Abdullah Mohammed Al‐Majid, M. Ali, Yaseen A. M. M. Elshaier, Hazem A. Ghabbour, Mohammad Shahidul Islam, Assem Barakat

**Affiliations:** ^1^ Department of Pharmacognosy, Faculty of pharmacy Mansura University Mansura 35516 Egypt; ^2^ Department of Chemistry, College of Science King Saud University P. O. Box 2455 Riyadh 11451 Saudi Arabia; ^3^ Department of Organic and Medicinal chemistry, Faculty of Pharmacy University of Sadat City Menoufiya Egypt; ^4^ Department of Medicinal Chemistry, Faculty of Pharmacy University of Mansoura Mansoura 35516 Egypt; ^5^ Department of Chemistry, Faculty of Science Alexandria University P.O. Box 426 Ibrahimia, Alexandria 21321 Egypt

**Keywords:** bimetallic catalysis, Lewis acid, Michael addition, indoles, barbituric acid, α-amylase, α-glucosidase, docking studies

## Abstract

The Michael addition reaction of barbituric acid with chalcones incorporating the indole scaffold was achieved by using a highly efficient bimetallic Iron–palladium catalyst in the presence of acetylacetone (acac). This catalytic approach produced the desired products in a simple operation and low catalyst loading with acceptable yield of the new hybrids. All tested compounds were subjected for biological activity on α‐glucosidase and α‐amylase. The results revealed that all synthesized compounds exhibited very good activity against both enzymes when compared to positive control (acarbose). Moreover, compound **5o** showed the best activity whereas its IC_50_ (*μ*M) are 13.02+0.01 and 21.71+0.82 for *α*‐glucosidase and *α*‐amylase respectively. Both compounds **5o** and **5l** exhibited high similarity in binding mode and pose with amylase protein (4UAC). The obtained data may be used for developing potential hypoglycemic agents.

## 1 Introduction

**Figure 1:**
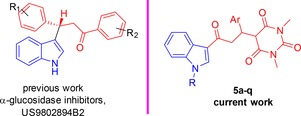
Previous and current study.

Heterocyclic compounds are of immense chemical and biological significance. In particular, azaheterocycles (nitrogen containing heterocycles) such as pyrimidines and indoles are structural constituents of many natural as well as synthetic bioactive drug‐like molecules.^1^ Substituted azaheterocycles have been referred as “privileged structures” since they are capable of binding to many receptors with high affinity and hydrogen bonding capacity. Naturally occurring nitrogen‐based heterocycles such as reserpine, vinca alkaloids, bisindoles, indoloquinolines, opioid analgesics, carbolines and cinchona alkaloids are established source of lead molecules for diverse therapeutic areas.^2^ Among the nitrogen containing heterocycles, indole is the parent core in a large number of bioactive naturally occurring compounds. Indole and its derivatives have received significant attention due to their wide range of biological activities including antimicrobial, anticancer, anti‐HIV antileishmanial and anti‐inflammatory.^3^ In recent past, several nitrogen containing novel chemical entities emerged as drug molecules, for example, Atevirdine (anti‐HIV); Camptothecin (CPT) (inhibitors of topoisomerase I);^4^ Cryptolepine (inhibitors topoisomerase II).^5^ Synthetic analogues of Cryptolepine such as IQDMA and benzo‐pyrido‐indole derivatives exhibited potent anticancer activity via interaction of DNA^6^. We are engaged in a research program for drug development as anti‐diabetes based on indole and pyrimdine scaffolds.^7^ One example of our invention the use of indole scaffold in the treatment and prevention of diabetes has been described (Figure 1).^7^, ^8^

Diabetes Mellitus (DM) is a growing global health concern. In 2017, diabetes affected an estimated 426 million adults people (20–79 years) world‐wide; by 2045 this numbers are expected to overrun 629 million.^9^ The release of free glucose from starch is mediated by two important enzymes: α‐amylase and α‐glucosidase. α‐Amylase is a metalloenzyme that cleaves polysaccharide chains, semi‐randomly creating shorter chains rapidly, whereas α‐glucosidase breaks these shorter chains into free glucose. The inhibition of these two enzymes can delay digestion, and absorption of carbohydrates, and hence, impair the postprandial hyperglycemia. Therefore, the aim of our work was to synthesize, through a Michael addition to a series of indole containing chalcones, new heterocycles that may act as inhibitors of these two enzymes

## 2 Results and Discussion

### 2.1 Synthesis

**Scheme 1:**
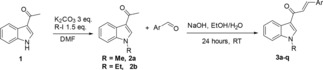
Synthesis of the chalcones **3a–q**.

The requisite compounds chalcones were prepared by reaction of N‐alkyl‐3‐acetylindole and aryl aldehyde derivatives stirring in EtOH/H_2_O (1 : 1) with NaOH at room temperature for 24 h. The product was produced in high yield (up to 90 %), as depicted in Scheme 1. The configuration of the chalcones obtained exclusively with *E*‐geometry. The *E* configuration of these compounds was supposed in analogy with similar compounds, previously prepared by us, whose configuration was established through X‐ray analysis.14b

Reaction of (*E*)‐1‐(1‐methyl‐1*H*‐indol‐3‐yl)‐3‐phenylprop‐2‐en‐1‐one **3a** with barbituric acid **4** was chosen as a model reaction to prepare 1,3‐dimethyl‐5‐(3‐(1‐methyl‐1*H*‐indol‐3‐yl)‐3‐oxo‐1‐phenylpropyl)pyrimidine‐2,4,6(1*H*,3*H*,5*H*)‐trione **5a**. Initially, the reaction of **3a** with barbituric acid **4** carried out in toluene at 80 °C in the presence of Cu(OTf)_2_/L1 (10 : 10 mol%) did not work all.^10^ However, upon using different solvents; THF, ACN, or Toluene/THF mixture, the reaction did not occur. Other metal salt as Zn(OTf)_2_ did not facilitate the reaction under the same conditions. Additionally, one attempt with FeCl_3_/PdCl_2_ carried out in MeOH at 60 °C, the reaction didnot occur at all.

Only, Fe−Pd bimetallic system^11^ in MeOH at 60 °C provides the desired product in moderate yield (55 %) (Table 1). The molecular structures of target compounds **5a** were determined by analysis of its spectroscopic data including ^1^H‐, ^13^C‐NMR, Fourier‐transform infra‐red (FT‐IR) spectroscopy and X‐ray crystal analysis.

**Table 1:** Model example for investigation of the reaction parameters.

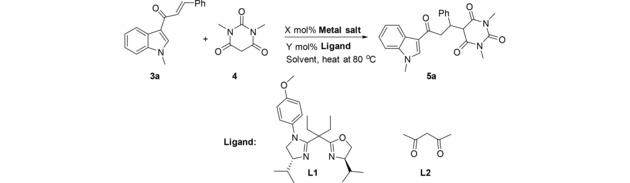					
#	Solvent	Metal Salts	Ligands	Ligand : Metal mol %	Yield
1.	Toluene	Cu(OTf)_2_	L1	10 : 11 mol%	No rxn^[b]^
2.	Toluene	Zn(OTf)_2_	L1	10 : 11 mol%	No rxn
3.	Toluene/THF	Zn(OTf)_2_	L1	10 : 11 mol%	No rxn
4.	THF	Zn(OTf)_2_	L1	10 : 11 mol%	No rxn
5.	ACN	Zn(OTf)_2_	L1	10 : 11 mol%	No rxn
6.	MeOH	FeCl_3_/PdCl_2_	L1	10 : 10 mol%	No rxn
7.	MeOH^[a]^	FeCl_3_/PdCl_2_	L2	10 : 10 mol%	55 %

[a] The reaction carried out at 60 °C. [b] No rxn: No reaction.

To investigate the generality of this method, the reaction of barbituric acid and different enones was examined under the optimized reaction conditions (10 mol% of FeCl_3_, 10 mol% of PdCl_2_ and 15 mol% Acac, 1.0 equiv. chalcone and 1.1 equiv. barbituric acid in CH_3_OH at at 60 °C. All of the results are summarized in Table 2.

**Table 2:** Substrate scope of desired compounds **5a–q**.

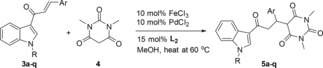					
#	Chlacones **3a–q**	Ar	R	Products **5a–q**	[%] Yield **5a–q**
1.	**3a**	Ph	Me	**5a**	**55**
2.	**3b**	4‐MePh	Et	**5b**	**44.9**
3.	**3c**	4‐ClPh	Et	**5c**	**60.2**
4.	**3d**	2,4‐Cl_2_Ph	Et	**5d**	**55.1**
5.	**3e**	4‐OMePh	Et	**5e**	**53**
6.	**3f**	4‐BrPh	Et	**5f**	**39.3**
7.	**3g**	4‐FPh	Et	**5g**	**47.6**
8.	**3h**	3‐FPh	Et	**5h**	**46.8**
9.	**3i**	3‐MePh	Et	**5i**	**46.6**
10.	**3j**	3‐BrPh	Et	**5j**	**36.7**
11.	**3k**	4‐CF_3_Ph	Et	**5k**	**39.7**
12.	**3l**	Thiophinyl	Et	**5l**	**53.7**
13.	**3m**	Furanyl	Et	**5m**	**54.6**
14.	**3n**	3,4,5‐OMe_3_Ph	Et	**5n**	**35.5**
15.	**3o**	2‐Napthyl	Et	**5o**	**37**
16.	**3p**	2,4,6‐Me_3_Ph	Et	**5p**	**‐**
17.	**3q**	4‐NO_2_Ph	Et	**5q**	**34.6**

### 2.2 X‐Ray Crystallography

**Figure 2:**
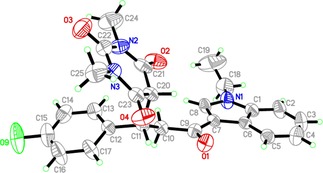
ORTEP diagram of the titled compounds **5g**. Displacement ellipsoids are plotted at the 40 % probability level for non‐H atoms.

**Figure 3:**
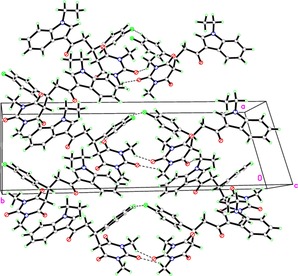
Molecular packing of titled compounds **5g** viewed hydrogen bonds which are drawn as dashed lines along b axis.

The structure of **5g** was further confirmed by X‐Ray structural study. The asymmetric unit contains one independent molecule that is shown in Figure 2. It was found to crystallize in Monoclinic Cc space group. The crystallographic data and refinement information are summarized in Table 3 and bond lengths are in normal ranges as shown in Table 4. The crystal structure reveals that the title compound is found in three planes, the angles between indole ring plane (C1−C8/N1) and fluorophenyl ring (C12−C17) and pyrimidine moiety (C20−C21−N2−C22−N3−C23) are 22.41° and 41.07°, respectively. The angle between fluorophenyl ring and pyrimidine ring is 57.88°. The crystal structure is stabilized by many non‐classical hydrogen bonds along the b axis direction Figure 3, Table 5.

**Table 3:** Experimental details of **5g**.

Crystal data **5 g**	
Chemical formula	C_25_H_24_FN_3_O_4_
*M*r	449.47
Crystal system, space group	Monoclinic, *Cc*
Temperature (K)	293
*a*, *b*, *c* (Å)	12.128 (5), 28.221 (12), 8.718 (3)
β (°)	129.532 (9)
*V* (Å^3^)	2301.4 (16)
*Z*	4
Radiation type	Mo *K*α radiation
μ (mm^−1^)	0.09
Crystal size (mm)	0.33×0.20×0.09

Data collection	
Diffractometer	Bruker APEX‐II D8 venture
Absorption correction	Multi‐scan, SADABS Bruker 2014
θ_max_	27.0°
No. of measured, independent and observed [I>2σ(I)] reflections	21063, 4914, 2093
*R*_int_	0.180

Refinement	
R[*F* ^*2*^>2σ( *F* ^*2*^)], wR( *F* ^*2*^), S	0.067, 0.184, 0.99
No. of reflections	4914
No. of parameters	302
No. of restraints	2
H‐atom treatment	H atoms treated by a mixture of independent and constrained refinement
Δρ_max_, Δρ_min_ (e Å^−3^)	0.22, −0.15
CCDC	1877313

**Table 4:** Selected geometric parameters (Å, °) of **5g**.

O9−C15	1.374 (16)	N1−C18	1.488 (10)
O1−C9	1.230 (10)	N2−C21	1.369 (12)
O2−C21	1.216 (9)	N2−C22	1.384 (11)
O3−C22	1.231 (11)	N2−C24	1.477 (14)
O4−C23	1.208 (9)	N3−C22	1.353 (11)
N1−C1	1.386 (11)	N3−C23	1.372 (11)
N1−C8	1.355 (10)	N3−C25	1.479 (10)
C1−N1−C8	108.3 (6)	O1−C9−C10	120.6 (7)
C1−N1−C18	126.0 (7)	O9−C15−C14	116.6 (10)
C8−N1−C18	125.7 (7)	O9−C15−C16	120.0 (13)
C21−N2−C22	123.7 (7)	N1−C18−C19	115.2 (9)
C21−N2−C24	119.2 (7)	O2−C21−N2	119.9 (7)
C22−N2−C24	117.0 (8)	O2−C21−C20	121.6 (8)
C22−N3−C23	124.4 (6)	N2−C21−C20	118.5 (7)
C22−N3−C25	117.5 (7)	O3−C22−N2	120.2 (9)
C23−N3−C25	118.1 (6)	O3−C22−N3	121.1 (8)
N1−C1−C2	129.6 (7)	N2−C22−N3	118.7 (8)
N1−C1−C6	107.6 (7)	O4−C23−N3	120.1 (7)
N1−C8−C7	110.8 (7)	O4−C23−C20	121.5 (8)
O1−C9−C7	121.3 (8)	N3−C23−C20	118.4 (7)

**Table 5:** Hydrogen‐bond geometry (Å, °) of **5g**.

*D*−H⋅⋅⋅*A*	*D*−H	H⋅⋅⋅*A*	*D*⋅⋅⋅*A*	*D*−H⋅⋅⋅*A*
C10−H10B⋅⋅⋅O2	0.9700	2.3200	2.975 (12)	124.00
C14−H14A⋅⋅⋅O9^i^	0.9300	2.3400	3.170 (13)	148.00
C18−H18A⋅⋅⋅O4^ii^	0.9700	2.4500	3.364 (14)	156.00
C25−H25B⋅⋅⋅O3^iii^	0.9600	2.5400	3.388 (15)	148.00

Symmetry codes: (**i**) x, −y+1, z+1/2; (**ii**) x+1/2, −y+1/2, z+3/2; (**iii**) x, −y+1, z−1/2.

### 2.3 Biological Activity

The present study seeks an alternative drug among series of synthesized compounds that can regulate the hyperglycemia by down‐regulating alpha‐glucosidase and alpha‐amylase activity by using virtual and in vitro assays.

The data reported in Table 6 showed that the most active compounds, both on α‐glucosidase and on α‐amylase, are compounds **5o**, **5k**, and **5l**. All other compounds were found to have only good to moderate activity ranging from 28.05+0.41 to 77.05+0.04 *μ*M in the case of *α*‐glucosidase, but in the range of 53.10+0.10 to 96.42+0.22 *μ*M in the case of *α*‐amylase. Structure activity relationship indicates the importance of the naphthyl moiety in **5o**, of the *p*‐CF_3_Ph propanone substituted indole in **5k**, and of a thiophene ring in **5l**. The most active compound is **5o**, which showed an IC_50_=13.02+0.01 *μ*M and 21.71+0.82 *μ*M, for α‐glucosidase, and α ‐ amylase respectively.

**Table 6:** Results of the *α*‐glucosidase and *α*‐Amylase inhibitory activity of the synthesized compounds **5a–q**.

#	Compounds	α‐Glucosidase	α‐Amylase
		IC_50_ (*μ*M)*	
1	**5a**	65.14±0.17	93.25±0.10
2	**5b**	53.15±0.12	80.17±0.05
3	**5c**	49.75±0.01	71.24±0.20
4	**5d**	58.21±0.09	96.42±0.22
5	**5e**	61.42±0.78	88.45±0.32
6	**5f**	53.15±0.12	78.25±0.10
7	**5g**	69.75±0.01	86.42±0.22
8	**5h**	61.10±0.42	89.45±0.44
9	**5i**	73.15±0.12	95.25±0.10
10	**5j**	77.05±0.04	86.42±0.22
11	**5k**	20.49±0.44	47.11±0.09
12	**5l**	22.28±0.48	35.42±0.60
13	**5m**	64.35±0.08	82.15±0.50
14	**5n**	53.15±0.12	93.25±0.10
15	**5o**	13.02±0.01	21.71±0.82
16	**5q**	31.12±0.11	63.00±0.61
STD	**Acarbose (μM)**	2.35±0.13	0.75±0.07

*α‐Glucosidase and ±‐amylase are expressed with mean±SD of triplicates.

### 2.4 Docking Studies

The compound **5 o** was selected for docking study with (4UAC) because of its strongest inhibitory activity among these derivatives. The X‐ray crystal structure of (4UAC) was obtained from protein data bank (PDB ID: 4UAC).^12^ Protein‐ligand docking was operated by (OpenEye Scientific Software, Santa Fe, NM 87508).^13^ The binding site of the protein was prepared by employing FRED RECEPTOR 2.2.5 (OpenEye Scientific Software, Santa Fe, NM 87508).

**Figure 4:**
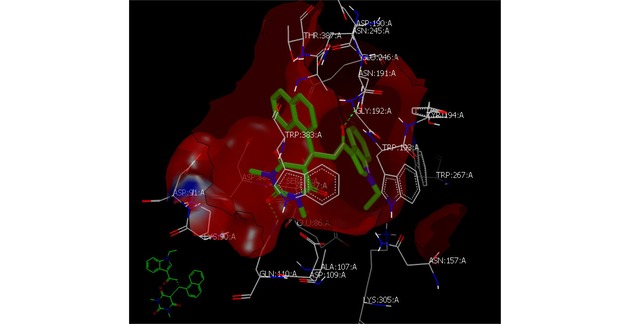
Snap shot visualization of **5o** docked with ID: **4AUC**, showing formation of two HBs interaction as illustrated by Vida

In the figure 4, we can find that compound **5o** formed hydrogen bonds to ASN 191 AA through the oxygen of carbonyl linked to indole moiety. Moreover, this compound formed another HB with GLN 110 AA through the carbonyl of barbiturate ring. These two interactions are similar to acrabose standard with receptor in its cocrystalized from.^12^

**Figure 5:**
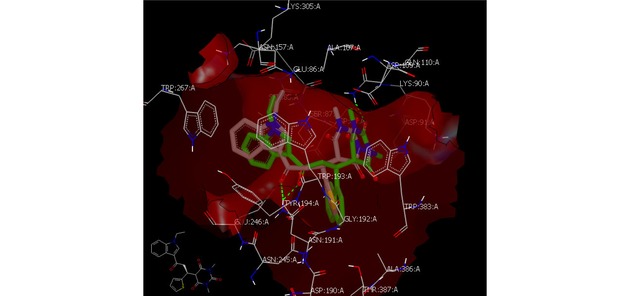
Snap shot visualization of compound **5l** overlays with **5o** and shown same binding mode and pose with receptor.

Compound **5o** exhibited high similarity to the potent derivative (compound **5l)** in the specific receptor, figure 5.

## Experimental Section

### General Procedure for the Synthesis of Chalcones 3a–q

The chalcones were prepared followed by reported procedure.^14^

#### (*E*)‐1‐(1‐Methyl‐1*H*‐indol‐3‐yl)‐3‐phenylprop‐2‐en‐1‐one (3a)

Yield 0.75 g (2.8 mmol, 53.8 %); All other spectral data are consistent with reported literature.14c

#### (*E*)‐1‐(1‐Ethyl‐1*H*‐indol‐3‐yl)‐3‐(*p–*tolyl)prop‐2‐en‐1‐one (3b)

Yield 1.34 g (4.63 mol, 86.8 %); m.p. 85–86 °C; ^1^H‐NMR (400 MHz, DMSO‐*d*_6_) *δ*: 1.47 (t, 3H, *J*=7.2 Hz, CH_3_), 2.29 (s, 3H, CH_3_) 4.12 (q, 2H, *J*=7.2 Hz, CH_2_), 7.12 (d, 2H, *J*=7.6 Hz, Ar−H), 7.22–7.28 (m, 4H, Ar−H & CH=CH), 7.45 (d, 2H, *J*=8.0 Hz, Ar−H), 7.71 (d, 1H, *J*=15.6 Hz, CH=CH), 7.81 (s, 1H, Ar−H), 8.44–8.46 (m, 1H, Ar−H); ^13^C‐NMR (100 MHz, DMSO‐*d*_6_) *δ*: 15.1, 21.4, 41.8, 109.6, 117.7, 122.5, 122.9, 123.0, 123.4, 127.0, 128.0, 129.5, 132.6, 133.5, 136.6, 104.0, 140.9, 184.4; IR (KBr, cm^−1^) ν_max_=3043, 2979, 1643, 1585, 1523, 1486, 1447, 1388, 1308, 1299, 1205, 1205, 1185, 1087; [Anal. Calcd. for C_20_H_19_NO: C, 83.01; H, 6.62; N, 4.84; Found: C, 83.41; H, 6.12; N, 4.32]; LC/MS (ESI, *m/z*): [M^+^], found 290.32, C_20_H_19_NOfor 289.15.

#### (*E*)‐3‐(4‐Chlorophenyl)‐1‐(1‐ethyl‐1*H*‐indol‐3‐yl)prop‐2‐en‐1‐one (3c)

Yield 1.56 g (5.04 mmol, 94.5 %); All other spectral data are consistent with reported literature.14d

#### (*E*)‐3‐(2,4‐Dichlorophenyl)‐1‐(1‐ethyl‐1*H*‐indol‐3‐yl)prop‐2‐en‐1‐one (3d)

Yield 1.70 g (4.9 mmol, 92.8 %); m.p. 168–169 °C; ^1^H‐NMR (400 MHz, DMSO‐*d*_6_) *δ*: 148 (t, 3H, *J*=7.2 Hz, CH_3_), 4.14 (q, 2H, *J*=7.2 Hz, CH_2_), 7.15–7.19 (m, 1H, Ar−H), 7.23–7.28 (m, 4H, Ar−H & CH=CH), 7.35 (d, 1H, *J*=2.4 Hz, Ar−H), 7.56 (d, 1H, *J*=8.0 Hz, Ar−H), 7.80 (s, 1H, Ar−H), 7.98 (d, 1H, *J*=15.2 Hz, CH=CH), 8.42–8.43 (m, 1H, Ar−H); ^13^C‐NMR (100 MHz, DMSO‐*d*_6_) *δ*: 15.3, 42.1, 109.8, 117.7, 122.9, 123.2, 123.9, 127.1, 127.5, 128.4, 130.1, 132.4, 134.1, 135.8, 135.8, 136.7, 184.1; IR (KBr, cm^−1^) ν_max_ = 3046, 2971, 2926, 2872, 1653, 1595, 1582, 1527, 1464, 1392, 1238, 1200, 1124, 1098, 1057; [Anal. Calcd. for C_19_H_15_Cl_2_NO: C, 66.29; H, 4.39; N, 4.07; Found: C, 66.42; H, 4.23; N, 4.36]; LC/MS (ESI, *m/z*): [M^+^], found 344.10, C_19_H_15_Cl_2_NO for 343.05.

#### (*E*)‐1‐(1‐Ethyl‐1*H*‐indol‐3‐yl)‐3‐(4‐methoxyphenyl)prop‐2‐en‐1‐one (3e)

Yield 1.60 g (5.2 mmol, 98.1 %); All other spectrum data are consistent with reported literature.14d

#### (*E*)‐3‐(4‐Bromophenyl)‐1‐(1‐ethyl‐1*H*‐indol‐3‐yl)prop‐2‐en‐1‐one (3f)

Yield 1.75 g (4.95 mmol, 92.8 %); m.p. 139–140 °C; ^1^H‐NMR (400 MHz, DMSO‐*d*_6_) *δ*: 1.49 (t, 3H, *J*=7.2 Hz, CH_3_), 4.17 (q, 3H, *J*=7.2 Hz, CH_2_), 7.23–7.29 (m, 4H, Ar−H & CH=CH), 7.41 (q, 4H, *J*=6.8 Hz, Ar−H), 7.64 (d, 1H, *J*=15.2 Hz, CH=CH), 7.82 (s, 1H, Ar−H), 8.42–8.45 (m, 1H, ArH); ^13^C‐NMR (100 MHz, DMSO‐*d*_6_) *δ*: 15.1, 41.8, 109.8, 117.6, 122.7, 123.0, 123.6, 123.8, 124.4, 126.9, 129.5, 131.9, 133.7, 133.3, 136.7, 139.5, 183.8; IR (KBr, cm^−1^) ν_max_=3449, 3041, 2977, 1645, 1610, 1588, 1525, 1481, 1469, 1454, 1399, 1310, 1241, 1267, 1268, 1016; [Anal. Calcd. for C_19_H_16_BrNO: C, 64.42; H, 4.55; N, 3.95; Found: C, 64.31; H, 4.67; N, 4.15]; LC/MS (ESI, *m/z*): [M^+^], found 354.18, C_19_H_16_BrNO for 353.04.

#### (*E*)‐1‐(1‐Ethyl‐1*H*‐indol‐3‐yl)‐3‐(4‐fluorophenyl)prop‐2‐en‐1‐one (3g)

Yield 1.40 g (4.77 mmol, 89.4 %); m.p. 94–95 °C; ^1^H‐NMR (400 MHz, DMSO‐*d*_6_) *δ*: 1.44 (t, 3H, *J*=7.2 Hz, CH_3_), 4.12 (q, 2H, *J*=7.2 Hz, CH_2_), 7.96–7.02 (m, 2H, Ar−H), 7.15–7.30 (m, 4H, Ar−H & CH=CH), 7.49 –7.54 (m, 2H, Ar−H), 7.65‐7.70 (m, 1H,CH=CH), 7.82 (s, 1H, Ar−H), 8.41–8.45 (m, 1H, Ar−H); ^13^C‐NMR (100 MHz, DMSO‐*d*_6_) *δ*: 15.1, 41.8, 109.8, 115.7, 115.9, 117.6, 122.6, 123.0, 123.5, 123.7, 126.8, 129.5, 131.6, 133.7, 136.7, 139.6, 164.8, 183.8; IR (KBr, cm^−1^) ν_max_=3451, 3047, 2979, 1642, 1613, 1589, 1524, 1482, 1468, 1450, 1397, 1313, 1242, 1269, 1262, 1015; [Anal. Calcd. for C_19_H_16_FNO: C, 77.80; H, 5.50; N, 4.77; Found: C, 78.05; H, 5.59; N, 4.61]; LC/MS (ESI, *m/z*): [M^+^], found 294.280, C_19_H_16_FNO for 293.12.

#### (*E*)*‐*1‐(1‐Ethyl‐1*H*‐indol‐3‐yl)‐3‐(3‐fluorophenyl)prop‐2‐en‐1‐one (3h)

Yield 1.40 g (4.77 mmol, 89.4 %); m.p. 84–85 °C; ^1^H‐NMR (400 MHz, DMSO‐*d*_6_) *δ*: 1.47 (t, 3H, *J*=6.0 Hz, CH_3_), 4.15 (q, 2H, *J*=6.0 Hz, CH_2_), 6.94–7.00 (m, 1H, Ar−H), 7.24–7.29 (m, 7H, *J*=6.8 Hz, Ar−H & CH=CH), 7.67 (d, 1H, *J*=15.2 Hz, CH=CH), 7.83 (s, 1H, Ar−H), 8.43–8.45 (m, 1H, ArH); ^13^C‐NMR (100 MHz, DMSO‐*d*_6_) *δ*: 15.3, 42.0,109.9, 114.0 & 114.2, 116.6 & 116.8, 117.7, 122.9, 123.2, 123.7, 124.4, 125.0, 127.0, 130.4 & 130.5, 133.9, 136.8, 137.7 & 137.8, 139.6, 162.4, 164.1, 184.8; IR (KBr, cm^−1^) ν_max_=3459, 3049, 2977, 1646, 1611, 1588, 1524, 1486, 1463, 1449, 1391, 1311, 1245, 1268, 1260, 1011; [Anal. Calcd. for C_19_H_16_FNO: C, 77.80; H, 5.50; N, 4.77; Found: C, 77.95; H, 5.37; N, 4.48]; LC/MS (ESI, *m/z*): [M^+^], found 294.21, C_19_H_16_FNO for 293.12.

#### (*E*)‐1‐(1‐Ethyl‐1*H*‐indol‐3‐yl)‐3‐(m–tolyl)prop‐2‐en‐1‐one (3i)

Yield 1.28 g (4.42 mmol, 82.9 %); m.p. 116–117 °C; ^1^H‐NMR (400 MHz, DMSO‐*d*_6_) *δ*: 1.54 (t, 3H, *J*=7.2 Hz, CH_3_), 2.38 (s, 3H, CH_3_), 4.22 (q, 2H, *J*=7.2 Hz, CH_2_), 7.17 (d, 1H, *J*=3.6 Hz, Ar−H), 7.24–7.47 (m, 7H, Ar−H & CH=CH), 7.78 (d, 1H, *J*=15.2 Hz, CH=CH), 7.9 (s, 1H, Ar−H), 8.50–8.53 (m, 1H, Ar−H); ^13^C‐NMR (100 MHz, DMSO‐*d*_6_) *δ*: 15.1, 21.3, 41.8, 109.7, 117.7, 122.6, 123.1, 123.4, 123.6, 125.3, 127.0, 128.6, 130.6, 133.7, 135.3, 136.7, 138.4, 138.4, 141.2, 184.3; IR (KBr, cm^−1^) ν_max_=3046, 2977, 1644, 1589, 1525, 1482, 1449, 1389, 1304, 1297, 1206, 1204, 1188, 1088; [Anal. Calcd. for C_20_H_19_NO: C, 83.01; H, 6.62; N, 4.84; Found: C, 83.35; H, 6.51; N, 4.73]; LC/MS (ESI, *m/z*): [M^+^], found 290.24, C_20_H_19_NO for 289.15.

#### (*E*)‐3‐(3‐Bromophenyl)‐1‐(1‐ethyl‐1*H*‐indol‐3‐yl)prop‐2‐en‐1‐one (3j)

Yield 1.60 g (4.53 mmol, 84.8 %); m.p. 126–127 °C; ^1^H‐NMR (400 MHz, DMSO‐*d*_6_) *δ*: 1.54 (t, 3H, *J*=5.6 Hz, CH_3_)„ 4.22 (q, 2H, *J*=5.6 Hz, CH_2_), 7.25–7.36 (m, 5H, Ar−H & CH=CH), 7.49 (t, 2H, *J*=8.8 Hz, Ar−H), 7.68–7.78 (m, 2H, *J*=15.2 Hz, Ar−H & CH=CH), 7.92 (s, 1H, Ar−H), 8.50–8.53 (m, 1H, Ar−H); ^13^C‐NMR (100 MHz, DMSO‐*d*_6_) *δ*: 15.3, 42.0, 109.9, 117.7, 122.9, 123.01, 123.2, 123.8, 125.1, 127.05, 127.3, 130.3, 130.4, 132.6, 134.03, 136.8, 137.6, 139.3, 183.7; IR (KBr, cm^−1^) ν_max_=3445, 3042, 2971, 1643, 1617, 1585, 1521, 1482, 1467, 1455, 1399, 1312, 1242, 1261, 1264, 1012; [Anal. Calcd. for C_19_H_16_BrNO: C, 64.42; H, 4.55 N, 3.95; Found: C, 64.65; H, 4.38; N, 4.17]; LC/MS (ESI, *m/z*): [M^+^ found 354.19, C_19_H_16_BrNO for 353.04.

#### (*E*)‐1‐(1‐Ethyl‐1*H*‐indol‐3‐yl)‐3‐(4‐(trifluoromethyl)phenyl)prop‐2‐en‐1‐one (3k)

Yield 1.7 g (4.9 mmol, 92.7 %); m.p. 150–151 °C; ^1^H‐NMR (400 MHz, DMSO‐*d*_6_) *δ*: 1.54 (t, 3H, *J*=7.2 Hz, CH_3_), 4.23 (q, 2H, *J*=7.2 Hz, CH_2_), 7.31–7.39 (m, 3H, Ar−H), 7.44 (d, 1H, *J*=15.2 Hz, CH=CH), 7.61 (d, 2H, *J*=8.0 Hz, Ar−H), 7.69 (d, 2H, *J*=8.0 Hz, Ar−H), 7.80 (d, 1H, *J*=15.2 Hz, CH=CH), 7.92 (s, 1H, Ar−H), 8.49–8.51 (m, 1H, Ar−H); ^13^C‐NMR (100 MHz, DMSO‐*d*_6_) *δ*: 15.1, 41.7, 109.7, 112.3, 114.6, 117.7, 121.4, 122.6, 123.1, 123.4, 126.9, 127.4, 133.8, 136.7, 144.03, 152.0, 183.8; IR (KBr, cm^−1^) ν_max_=3441, 3037, 2981, 1652, 1624, 1596, 1532, 1475, 1471, 1456, 1388, 1327, 1246, 1275, 1252, 1011; [Anal. Calcd. for C_20_H_16_F_3_NO: C, 69.96; H, 4.70; N, 4.08; Found: C, 70.12; H, 4.92; N, 4.40]; LC/MS (ESI, *m/z*): [M^+^], found 344.24, C_20_H_16_F_3_NO for 343.12

#### (*E)‐*1‐(1‐Ethyl‐1*H*‐indol‐3‐yl)‐3‐(thiophen‐2‐yl)prop‐2‐en‐1‐one (3l)

Yield 1.46 g (5.19 mmol, 97.2 %); m.p. 119–120 °C; ^1^H‐NMR (400 MHz, DMSO‐*d*_6_) *δ*: 1.47 (t, 3H, *J*=7.6 Hz, CH_3_), 4.14 (q, 2H, *J*=7.6 Hz, CH_2_), 6.98 (t, 1H, *J*=7.6 Hz, Ar−H), 7.10 (d, 1H, *J*=15.6 Hz, CH=CH), 7.24–7.30 (m, 5H, Ar−H), 7.81 (s, 1H, Ar−H), 7.86 (d, 1H, *J*=15.6 Hz, CH=CH), 8.45–8.44 (m, 1H, Ar−H); ^13^C‐NMR (100 MHz, DMSO‐*d*_6_) *δ*: 15.1, 41.8, 109.7, 117.6, 122.6, 122.8, 123.1, 123.5, 126.9, 127.5, 128.1, 130.9, 133.6, 133.7, 136.7, 140.8, 183.6; IR (KBr, cm^−1^) ν_max_=3474, 3106, 3073, 2970, 2928, 1632, 1560, 1522, 1486, 1447, 1388, 1360, 1207, 1103, 1085,; [Anal. Calcd. for C_17_H_15_NOS: C, 72.57; H, 5.37; N, 4.98; Found: C, 72.82; H, 5.15; N, 5.10]; LC/MS (ESI, *m/z*): [M^+^], found 282.23, C_17_H_15_NOS for 281.09.

#### (*E*)‐1‐(1‐Ethyl‐1*H*‐indol‐3‐yl)‐3‐(furan‐2‐yl)prop‐2‐en‐1‐one (3m)

Yield 1.35 g (5.09 mmol, 95.4 %); m.p. 74–75 °C; ^1^H‐NMR (400 MHz, DMSO‐*d*_6_) *δ*: 1.54 (t, 3H, *J*=7.2 Hz, CH_3_), 4.23 (q, 2H, *J*=7.2 Hz, CH_2_), 7.32–7.37 (m, 3H, Ar−H), 7.42 (d, 1H, *J*=15.6 Hz, CH=CH), 7.61 (d, 2H, *J*=8.0 Hz, Ar−H), 7.69 (d, 2H, *J*=8.0 Hz, Ar−H), 7.75 (d, 1H, *J*=15.2 Hz, CH=CH), 7.92 (s, 1H, Ar−H ), 8.49–8.51 (m, 1H, Ar−H); ^13^C‐NMR (100 MHz, DMSO‐*d*_6_) *δ*: 15.1, 41.7, 109.7, 112.3, 114.6, 117.7, 121.3, 123.0, 123.4, 126.9, 127.4, 133.8, 136.7, 144.0, 152.0, 183.7; IR (KBr, cm^−1^) ν_max_=3478, 3102, 3075, 2972, 2929, 1631, 1562, 1524, 1488, 1442, 1389, 1365, 1204, 1102, 1086; [Anal. Calcd. for C_17_H_15_NO_2_: C, 76.96; H, 5.70; N, 5.28; Found: C, 76.55; H, 5.95; N, 5.10]; LC/MS (ESI, *m/z*): [M^+^], found 266.27, C_17_H_15_NO_2_ for 265.11.

#### (*E*)‐1‐(1‐Ethyl‐1*H*‐indol‐3‐yl)‐3‐(3,4,5‐trimethoxyphenyl)prop‐2‐en‐1‐one (3n)

Yield 1.1 g (3.01 mmol, 56.4 %); m.p. 197–198 °C; ^1^H‐NMR (400 MHz, DMSO‐*d*_6_) *δ*: 1.53 (t, 3H, *J*=7.6 Hz, CH_3_), 3.87 (s, 3H, OCH_3_) 3.90 (s, 6H, OCH_3_), 4.23 (q, 2H, *J*=7.6 Hz, CH_2_), 6.83 (s, 2H, Ar−H), 7.26 (d, 1H, *J*=15.2 Hz, CH=CH), 7.30–7.37 (m, 3H, Ar−H), 7.71 (d, 1H, *J*=15.2 Hz, CH=CH), 7.93 (s, 1H, Ar−H), 8.49–8.51 (m, 1H, Ar−H); ^13^C‐NMR (100 MHz, DMSO‐*d*_6_) *δ*: 15.2, 41.8, 56.2, 60.9, 105.4, 109.7, 117.6, 122.6, 123.1, 123.2, 123.5, 127.0, 130.9, 133.8, 136.8, 141.3, 153.4, 184.1; IR (KBr, cm^−1^) ν_max_=3452, 3103, 2977, 2942, 2831, 1639, 1581, 1566, 1522, 1463, 1447, 1419, 1392, 1337, 1250, 1147, 1121, 1002; [Anal. Calcd. for C_22_H_23_NO_4_: C, 72.31; H, 6.34; N, 3.83; Found: C, 72.46; H, 6.12; N, 3.51]; LC/MS (ESI, *m/z*): [M^+^], found 366.20, C_22_H_23_NO_4_ for 365.16.

#### (*E*)‐1‐(1‐Ethyl‐1*H*‐indol‐3‐yl)‐3‐(naphthalen‐2‐yl)prop‐2‐en‐1‐one (3o)

Yield 0.9 g (2.7 mmol, 51.8 %); m.p. 113–114 °C; ^1^H‐NMR (400 MHz, DMSO‐*d*_6_) *δ*: 1.46 (t, 3H, *J*=7.2 Hz, CH_3_), 4.14 (q, 2H, *J*=7.2 Hz, CH_2_), 7.23–7.28 (m, 3H, Ar−H), 7.38 –7.42 (m, 3H, Ar−H & CH=CH), 7.69 –7.78 (m, 4H, Ar−H), 7.89 (d, 3H, *J*=15.2 Hz, CH=CH & Ar−H), 8.46–8.48 (m, 1H, Ar−H); ^13^C‐NMR (100 MHz, DMSO‐*d*_6_) *δ*: 15.1, 41.8, 109.7, 117.7, 122.6, 123.1, 123.5, 123.8, 123.9, 126.5, 126.9, 127.0, 127.7, 128.4, 129.8, 132.8, 133.4, 133.9, 134.0, 136.7, 141.1, 184.1; IR (KBr, cm^−1^) ν_max_=3478, 3105, 3049, 2968, 2926, 2879, 1642, 1578, 1505, 1468, 1388, 1294, 1208, 1141, 1085; [Anal. Calcd. for C_23_H_19_NO: C, 84.89; H, 5.89; N, 4.30; Found: C, 84.96; H, 6.11; N, 4.62]; LC/MS (ESI, *m/z*): [M^+^], found 326.10, C_23_H_19_NO for 325.15.

#### (*E*)‐1‐(1‐Ethyl‐1*H*‐indol‐3‐yl)‐3‐mesitylprop‐2‐en‐1‐one (3p)

Yield 1.10 g (3.4 mmol, 64.9 %); m.p. 83–84 °C; ^1^H‐NMR (400 MHz, DMSO‐*d*_6_) *δ*: 1.52 (t, 3H, *J*=7.6 Hz, CH_3_), 2.29 (s, 3H, CH_3_) 2.36 (s, 6H, CH_3_), 4.22 (q, 2H, *J*=7.6 Hz, CH_2_), 6.91 (s, 2H, Ar−H), 6.99 (d, 1H, *J*=16.4 Hz, CH=CH), 7.30–7.38 (m, 3H, Ar−H), 7.80 (s, 1H, Ar−H), 7.93 (d, 1H, *J*=16.4 Hz, CH=CH), 8.50–8.53 (m, 1H, Ar−H); ^13^C‐NMR (100 MHz, DMSO‐*d*_6_) *δ*: 15.3, 21.1, 21.3,41.9, 109.8, 117.8, 122.7, 123.2, 123.6, 127.1, 129.1, 129.3, 132.3, 133.8, 136.8, 136.9, 137.9, 139.6, 184.5; IR (KBr, cm^−1^) ν_max_=3041, 2974, 1641, 1584, 1525, 1483, 1447, 1385, 1302, 1294, 1206, 1188, 1089, 1062; [Anal. Calcd. for C_22_H_23_NO: C, 83.24; H, 7.30; N, 4.41; Found: C, 83.52; H, 7.19; N, 4.61]; LC/MS (ESI, *m/z*): [M^+^], found 318.20; C_22_H_23_NO for 317.18.

#### (*E*)‐1‐(1‐Ethyl‐1*H*‐indol‐3‐yl)‐3‐(4‐nitrophenyl)prop‐2‐en‐1‐one (3q)

Yield 0.95 g (2.9 mmol, 55.5 %); m.p. 179–180 °C; ^1^H‐NMR (400 MHz, DMSO‐*d*_6_) *δ*: 1.45 (t, 3H, *J*=7.2 Hz, CH_3_), 4.1 (t, 2H, *J*=7.2 Hz, CH_2_), 7.24–7.30 (m, 5H, Ar−H & CH=CH), 7.55 (d, 2H, *J*=8.8 Hz, Ar−H), 8.13 (d, 2H, *J*=8.8 Hz, Ar−H), 8.17 (d, 1H, *J*=15.2 Hz, CH=CH), 8.25 ‐ 8.27 (m, 1H, Ar−H); ^13^C‐NMR (100 MHz, DMSO‐*d*_6_) *δ*: 15.1, 41.9, 109.9, 116.2, 122.5, 123, 123.6, 126.5, 128.5, 134.3, 136.6, 137.9, 141.7, 147.2, 150.8, 194.1; IR (KBr, cm^−1^) ν_max_=3472, 3116, 3069, 3049, 2973, 1744, 1679, 1638, 1529, 1462, 1423, 1378, 1285, 1205, 1129, 1110, 1052; [Anal. Calcd. for C_19_H_16_N_2_O_3_: C, 71.24; H, 5.03; N, 8.74; Found: C, 71.51; H, 5.19; N, 8.95]; LC/MS (ESI, *m/z*): [M^+^], found 321.19, C_19_H_16_N_2_O_3_ for 320.12.

### General Procedure for the Preparation of 5a‐q

FeCl_3_ (0.025 mmol), PdCl_2_ (0.025 mmol), and acetylacetone (0.075 mmol) were added into a solution of enone (0.5 mmol) and barbituric acid (0.55 mmol) in freshly distilled CH_3_OH (2 ml). After stirring at room temperature for 24 h, the mixture was diluted with H_2_O (10 ml) and extracted with EtOAc (3×15 ml). The combined organic layers were dried (Na_2_SO_4_), concentrated *in vacuo* and purified by column chromatography on silica gel (200–300 mesh, gradient eluted with EtOAc–petroleum ether=1 : 10–1: 5) to gain the pure product.

#### 1,3‐Dimethyl‐5‐(3‐(1‐methyl‐1*H*‐indol‐3‐yl)‐3‐oxo‐1‐phenylpropyl)pyrimidine‐2,4,6(1*H*,3*H*,5*H*)‐trione (5a)

Yield 230 mg (0.55 mmol, 55 %); m.p. 185–186 °C; ^1^H‐NMR (600 MHz, CDCl_3_) *δ*: 3.06 (s, 3H, NCH_3_), 3.11 (s, 3H, NCH_3_), 3.34–3.36 (dd, 1H, *J*=11.2 Hz, 3.6 Hz, CH_2(a)_), 3.87 (s, 3H, NCH_3_), 3.96–4.00 (m, 2H, CH_2(e)_& CH), 4.49–4.46 (m, 1H, CH), 2.48–2.58 (m, 1H, CH_2_), 3.82–3.92 (m, 1H, CHN), 7.11–7.13 (m, 2H, Ar−H), 7.26–7.35 (m, 6H, Ar−H), 7.93 (s, 1H,Ar−H), 8.34–8.39 (m, 1H, Ar−H); ^13^C‐NMR (150 MHz, CDCl_3_) *δ*: 28.0, 28.1, 33.6, 41.0, 44.9, 53.2, 109.6, 116.5, 122.6, 122.7, 123.4, 126.2, 127.3, 128.2, 128.6, 135.7, 137.4, 138.4, 151.0, 167.9, 168.3, 192.2; IR (KBr, cm^−1^) ν_max_=3439, 3111, 3108, 3056, 2951, 1679, 1637, 1536, 1530, 1442, 1425, 1375, 1335, 1223, 1145, 1081; [Anal. Calcd. for C_24_H_23_N_3_O_4_: C, 69.05; H, 5.55; N, 10.07; Found: C, 69.23; H, 5.41; N, 9.95]; LC/MS (ESI, *m/z*): [M^+^], found 418.20, C_24_H_23_N_3_O_4_ for 417.17.

#### 1,3‐Dimethyl‐5‐(3‐(1‐methyl‐1*H*‐indol‐3‐yl)‐3‐oxo‐1‐(*p–*tolyl)propyl)pyrimidine‐2,4,6(1*H*,3*H*,5*H*)‐trione (5b)

Yield 200 mg (0.45 mmol, 44.9 %); m.p. 155–156 °C; ^1^H‐NMR (600 MHz, CDCl_3_) *δ*: 1.55 (t, 3H, *J*=4.2 Hz, NCH_2_CH_3_), 2.29 (s, 3H, CH_3_), 3.07 (s, 3H, NCH_3_), 3.11 (s, 3H, NCH_3_), 3.32–3.34 (dd, 1H, *J*=11.2 Hz, 3.6 Hz, CH_2(a)_), 3.95–3.99 (m, 2H, CH_2(e)_& CH), 4.23 (q, 2H, *J*=4.8 Hz, NCH_2_CH_3_), 4.38–4.44 (m, 1H, CH), 7.01 (d, 2H, *J*=5.6 Hz, Ar−H), 7.06 (d, 2H, *J*=5.2 Hz, Ar−H), 7.28–7.31 (m, 2H, Ar−H), 7.36–7.38 (m, 1H, Ar−H), 7.97 (s, 1H,Ar−H), 8.39–8.40 (m, 1H, Ar−H); ^13^C‐NMR (150 MHz, CDCl_3_) *δ*: 15.2, 21.1, 28.0, 28.1, 41.2, 41.8, 44.6, 53.3, 109.7, 116.5, 122.6, 122.8, 123.3, 126.5, 127.2, 129.3, 134.1, 135.4, 136.4, 137.9, 151.1, 168.0, 148.4, 192.4; IR (KBr, cm^−1^) ν_max_=3437, 3114, 3101, 3059, 2955, 1678, 1636, 1539, 1448, 1426, 1371, 1335, 1227, 1142, 1084; [Anal. Calcd. for C_26_H_27_N_3_O_4_: C, 70.09; H, 6.11; N, 9.43; Found: C, 69.87; H, 5.95; N, 9.63]; LC/MS (ESI, *m/z*): [M^+^], found 446.28, C_26_H_27_N_3_O_4_ for 445.20.

#### 5‐(1‐(4‐Chlorophenyl)‐3‐(1‐ethyl‐1*H*‐indol‐3‐yl)‐3‐oxopropyl)‐1,3‐dimethylpyrimidine‐2,4,6(1*H*,3*H*,5*H*)‐trione (5c)

Yield 280 mg (0.60 mmol, 60.2 %); m.p. 166–167 °C; ^1^H‐NMR (600 MHz, CDCl_3_) *δ*: 1.55 (t, 3H, *J*=4.2 Hz, NCH_2_CH_3_), 3.11 (s, 3H, NCH_3_), 3.16 (s, 3H, NCH_3_), 3.35–3.37(dd, 1H, *J*=11.2 Hz, 5.0 Hz, CH_2(a)_), 3.96 (t, 1H, *J*=6.4 Hz, CH_2(e)_), 3.98 (d, 1H, *J*=2.8 Hz, CH), 4.23 (q, 2H, *J*=4.8 Hz, NCH_2_CH_3_), 4.44–4.49 (m, 1H, CH), 7.12 (d, 2H, *J*=5.6 Hz, Ar−H), 7.24 (d, 2H, *J*=5.6 Hz, Ar−H), 7.29–7.31 (m, 2H, Ar−H), 7.37–7.39 (m, 1H, Ar−H), 7.95 (s, 1H,Ar−H), 8.33–8.37 (m, 1H, Ar−H); ^13^C‐NMR (150 MHz, CDCl_3_) *δ*: 15.2, 27.3, 28.4, 41.1, 41.9, 43.6, 53.1, 109.8, 116.5, 121.8, 122.7, 123.4, 126.5, 129.1, 129.2, 134.1, 135.2, 136.7, 137.8, 151.0, 167.3, 168.0, 192.3; IR (KBr, cm^−1^) ν_max_=3443, 2983, 1745, 1692, 1685, 1651, 1531, 1466, 1428, 1375, 1289, 1205, 1109, 1057; [Anal. Calcd. for C_25_H_24_ClN_3_O_4_: C, 64.44; H, 5.19; Cl, 7.61; N, 9.02; Found: C, 64.53; H, 5.32; N, 9.21]; LC/MS (ESI, *m/z*): [M^+^], found 466.20, C_25_H_24_ClN_3_O_4_ for 465.15.

#### 5‐(1‐(2,4‐Dichlorophenyl)‐3‐(1‐ethyl‐1*H*‐indol‐3‐yl)‐3‐oxopropyl)‐1,3‐dimethylpyrimidine‐2,4,6(1*H*,3*H*,5*H*)‐trione (5d)

Yield 275 mg (0.55 mmol, 55.1 %); m.p. 149–150 °C; ^1^H‐NMR (600 MHz, CDCl_3_) *δ*: 1.54 (t, 3H, *J*=4.8 Hz, NCH_2_CH_3_), 3.20 (s, 3H, NCH_3_), 3.22 (s, 3H, NCH_3_), 3.37–3.39 (dd, 1H, *J*=10.8 Hz, 4.0 Hz, CH_2(a)_), 3.38–3.71 (dd, 1H, *J*=10.8 Hz, 6.4 Hz, CH_2(a)_), 3.86 (d, 1H, *J*=2.4 Hz, CH), 4.23 (q, 2H, *J*=4.8 Hz, NCH_2_CH_3_), 4.90–4.93 (m, 1H, CH), 7.20 & 7.23 (dd, 1H, *J*=5.6 Hz, 1.6 Hz, Ar−H), 7.28–7.32 (m, 2H, Ar−H), 7.35–7.38 (m, 2H, Ar−H), 7.40 (d, 1H, *J*=1.6 Hz, Ar−H), 7.85 (s, 1H,Ar−H), 7.28 (d, 1H, *J*=4.8 Hz, Ar−H); ^13^C‐NMR (150 MHz, CDCl_3_) *δ*: 15.2, 28.2, 29.5, 38.9, 41.0, 42.1, 52.9, 109.8, 114.9, 122.6, 122.9, 123.2, 123.5, 127.2, 128.4, 129.8, 130.0, 133.9, 134.9, 135.9, 137.0, 150.1, 167.9, 168.4, 192.1; IR (KBr, cm^−1^) ν_max_=3449, 29.80, 17.47, 1694, 1681, 1653, 1530, 1461, 1427, 1379, 1288, 1201, 1104, 1052;[Anal. Calcd. for C_25_H_23_Cl_2_N_3_O_4_: C, 60.01; H, 4.63; N, 8.40; Found: C, 69.89; H, 4.71; N, 8.32]; LC/MS (ESI, *m/z*): [M^+^], found 500.21, C_25_H_23_Cl_2_N_3_O_4_ for 499.11.

#### 5‐(3‐(1‐Ethyl‐1*H*‐indol‐3‐yl)‐1‐(4‐methoxyphenyl)‐3‐oxopropyl)‐1,3‐dimethylpyrimidine‐2,4,6(1*H*,3*H*,5*H*)‐trione (5e)

Yield 245 mg (0.53 mmol, 53.1 %); m.p. 128–129 °C; ^1^H‐NMR (600 MHz, CDCl_3_) *δ*: 1.55 (t, 3H, *J*=5.2 Hz, NCH_2_CH_3_), 3.09 (s, 3H, NCH_3_), 3.12 (s, 3H, NCH_3_), 3.31–3.34 (dd, 1H, *J*=11.2 Hz, 3.6 Hz, CH_2(a)_), 3.76 (s, 3H, OCH_3_), 3.93–3.98 (m, 2H, CH_2(e)_& CH), 4.23 (q, 2H, *J*=4.8 Hz, NCH_2_CH_3_), 4.39–4.44 (m, 1H, CH), 6.85 (d, 2H, *J*=6.0 Hz, Ar−H), 7.50 (d, 2H, *J*=6.0 Hz, Ar−H), 7.28–7.31 (m, 2H, Ar−H), 7.36–7.38 (m, 1H, Ar−H), 7.97 (s, 1H,Ar−H), 8.38–8.41 (m, 1H, Ar−H); ^13^C‐NMR (150 MHz, CDCl_3_) *δ*: 15.2, 28.0, 28.2, 41.4, 41.8, 44.2, 53.4, 55.2, 109.7, 113.9, 116.6, 122.6, 122.7, 123.3, 126.5, 128.5, 130.4, 134.1, 136.5, 151.0, 159.2, 168.0, 168.5, 192.0; IR (KBr, cm^−1^) ν_max_=3434, 3103, 2985, 2947, 2835, 1749, 1673, 1647, 1582, 1523, 1516, 1421, 1374, 1331, 1241, 1208, 1126, 1101, 1006; [Anal. Calcd. for C_26_H_27_N_3_O_5_: C, C, 67.66; H, 5.90; N, 9.10; Found: C, 66.71; H, 5.97; N, 9.21]; LC/MS (ESI, *m/z*): [M^+^], found 462.30, C_26_H_27_N_3_O_5_ for 461.20.

#### 5‐(1‐(4‐Bromophenyl)‐3‐(1‐ethyl‐1*H*‐indol‐3‐yl)‐3‐oxopropyl)‐1,3‐dimethylpyrimidine‐2,4,6(1*H*,3*H*,5*H*)‐trione (5f)

Yield 200 mg (0.39 mmol, 39.3 %); m.p. 159–160 °C; ^1^H‐NMR (600 MHz, CDCl_3_) *δ*: 1.55 (t, 3H, *J*=5.2 Hz, NCH_2_CH_3_), 3.12 (s, 3H, NCH_3_), 3.16 (s, 3H, NCH_3_), 3.34–3.37 (dd, 1H, *J*=11.2 Hz, 4.0 Hz, CH_2(a)_), 3.96 & 3.98 (dd, 1H, *J*=11.2 Hz, 6.4 Hz, CH_2(e)_), 3.98 (d, 1H, *J*=2.4 Hz, CH), 4.23 (q, 2H, *J*=4.8 Hz, NCH_2_CH_3_), 4.44–4.46 (m, 1H, CH), 7.06 (d, 2H, *J*=5.6 Hz, Ar−H), 7.29–7.31 (m, 2H, Ar−H), 7.37–7.39 (m, 1H, Ar−H), 7.40 (d, 2H, *J*=5.6 Hz, Ar−H), 7.94 (s, 1H,Ar−H), 8.35–8.36 (m, 1H, Ar−H); ^13^C‐NMR (150 MHz, CDCl_3_) *δ*: 15.2, 28.2, 28.3, 41.2, 41.8, 43.6, 52.8, 109.8, 116.5, 122.0, 122.6, 122.7, 123.4, 126.4, 129.3, 131.8, 134.0, 134.5, 138.1, 150.9, 167.8, 168.0, 192.1; IR (KBr, cm^−1^) ν_max_=3452, 3116, 3043, 2974, 1744, 1679, 1638, 1526, 1462, 1423, 1378, 1285, 1205, 1110, 1052, 1009; [Anal. Calcd. for C_25_H_24_BrN_3_O_4_: C, 58.83; H, 4.74; N, 8.23; Found: C, 59.11; H, 4.59; N, 8.33]; LC/MS (ESI, *m/z*): [M^+^], found 510.17, C_25_H_24_BrN_3_O_4_ for 509.10.

#### 5‐(3‐(1‐Ethyl‐1H‐indol‐3‐yl)‐1‐(4‐fluorophenyl)‐3‐oxopropyl)‐1,3‐dimethylpyrimidine‐2,4,6(1*H*,3*H*,5*H*)‐trione (5g)

Yield 214 mg (0.48 mmol, 47.6 %); m.p. 185–186 °C; ^1^H‐NMR (600 MHz, CDCl_3_) *δ*: 1.55 (t, 3H, *J*=5.2 Hz, NCH_2_CH_3_), 3.10 (s, 3H, NCH_3_), 3.14 (s, 3H, NCH_3_), 3.35–3.38 (dd, 1H, *J*=11.2 Hz, 3.6 Hz, CH_2(a)_), 3.94–3.99 (m, 2H,CH_2(e)_& CH), 4.23 (q, 2H, *J*=4.8 Hz, NCH_2_CH_3_), 4.45–4.59 (m, 1H, CH), 6.96 (t, 2H, *J*=4.0 Hz, Ar−H), 7.15 (t, 2H, *J*=4.0 Hz, Ar−H), 7.27–7.31 (m, 2H, Ar−H), 7.37–7.39 (m, 1H, Ar−H), 7.96 (s, 1H,Ar−H), 8.37–8.38 (m, 1H, Ar−H); ^13^C‐NMR (150 MHz, CDCl_3_) *δ*: 15.2, 28.11, 28.3, 41.4, 41.7, 43.7, 53.2, 109.8, 115.5, 115.7, 121.9, 122.7, 123.4, 127.3, 129.1, 129.2, 134.1, 136.5, 139.1, 149.0, 167.0, 168.2, 191.2; IR (KBr, cm^−1^) ν_max_=3471, 3118, 2951, 1745, 1682, 1639, 1614, 1588, 1528, 1463, 1445, 1420, 1375, 1273, 1206, 1114, 1053; [Anal. Calcd. for C_25_H_24_FN_3_O_4_: C, 66.80; H, 5.38; N, 9.35; Found: C, 67.02; H, 5.54; N, 9.47]; LC/MS (ESI, *m/z*): [M^+^], found 450.20, C_25_H_24_FN_3_O_4_ for 449.18.

#### 5‐(3‐(1‐Ethyl‐1H‐indol‐3‐yl)‐1‐(3‐fluorophenyl)‐3‐oxopropyl)‐1,3‐dimethylpyrimidine‐2,4,6(1*H*,3*H*,5*H*)‐trione (5h)

Yield 210 mg (0.47 mmol, 46.8 %); m.p. 188–189 °C; ^1^H‐NMR (600 MHz, CDCl_3_) *δ*: 1.55 (t, 3H, *J*=5.2 Hz, NCH_2_CH_3_), 3.11 (s, 3H, NCH_3_), 3.15 (s, 3H, NCH_3_), 3.37–3.40 (dd, 1H, *J*=11.2 Hz, 3.6 Hz, CH_2(a)_), 3.95–3.98 (m, 2H,CH_2(e)_& CH), 4.22–4.26 (m, 2H CH), 4.24 (q, 2H, *J*=4.8 Hz, NCH_2_CH_3_), 4.45–4.59 (m, 1H, CH), 6.89–6.92 (m, 1H,Ar−H), 6.94–6.98 (m, 2H, Ar−H), 7.23–7.25 (m, 1H, Ar−H), 7.29–7.31 (m, 2H, Ar−H), 7.37–7.39 (m, 1H, Ar−H), 7.96 (s, 1H,Ar−H), 8.36–8.38 (m, 1H, Ar−H); ^13^C‐NMR (150 MHz, CDCl_3_) *δ*: 15.3, 28.2, 28.4, 41.1, 41.9, 44.1, 35.1, 109.8, 115.1, 115.2, 121.0, 122.7, 122.9, 123.5, 127.0, 129.5, 130.3, 134.2, 136.6, 137.9, 138.7, 150.2, 167.9, 168.3, 191.1; IR (KBr, cm^−1^) ν_max_=3471, 3118, 2951, 1745, 1682, 1639, 1614, 1588, 1528, 1463, 1445, 1420, 1375, 1273, 1206, 1114, 1053; [Anal. Calcd. for C_25_H_24_FN_3_O_4_: C, 66.80; H, 5.38; N, 9.35; Found: C, 67.13; H, 5.61; N, 9.41]; LC/MS (ESI, *m/z*): [M^+^], found 450.24, C_25_H_24_FN_3_O_4_ for 449.18.

#### 5‐(3‐(1‐Ethyl‐1*H*‐indol‐3‐yl)‐3‐oxo‐1‐(*m–*tolyl)propyl)‐1,3‐dimethylpyrimidine‐2,4,6(1*H*,3*H*,5*H*)‐trione (5i)

Yield 194 mg (0.44 mmol, 46.6 %); m.p. 125–126 °C; ^1^H‐NMR (600 MHz, CDCl_3_) *δ*: 1.56 (t, 3H, *J*=5.2 Hz, NCH_2_CH_3_), 2.29 (s, 3H, CH_3_), 3.06 (s, 3H, NCH_3_), 3.10 (s, 3H, NCH_3_), 3.34–3.37 (dd, 1H, *J*=11.2 Hz, 3.6 Hz, CH_2(a)_), 3.94–3.99 (m, 2H, CH_2(e)_& CH), 4.24 (q, 2H, *J*=4.8 Hz, NCH_2_CH_3_), 4.37–4.49 (m, 1H, CH), 6.90 (d, 1H, *J*=5.2 Hz, Ar−H), 6.93 (s, 1H, Ar−H), 7.06 (d, 1H, *J*=5.2 Hz, Ar−H), 7.15 (t, 1H, *J*=5.2 Hz, Ar−H), 7.29–7.31 (m, 2H, Ar−H), 7.36–7.38 (m, 1H, Ar−H), 7.98 (s, 1H,Ar−H), 8.39–8.41 (m, 1H, Ar−H); ^13^C‐NMR (150 MHz, CDCl_3_) *δ*: 15.2, 21.3, 27.9, 28.1, 41.0, 41.8, 45.0, 53.4, 109.7, 116.6, 122.6, 122.7, 123.3, 124.3, 126.5, 128.1, 128.4, 128.9, 134.1, 136.5, 138.4, 151.0, 168.0, 168.5, 192.3; IR (KBr, cm^−1^) ν_max_=3438, 3113, 3104, 3058, 2954, 1677, 1632, 1534, 1447, 1427, 1372, 1336, 1225, 1143, 1081; [Anal. Calcd. for C_26_H_27_N_3_O_4_: C, 70.09; H, 6.11; N, 9.43; Found: C, 70.29; H, 6.33; N, 9.57]; LC/MS (ESI, *m/z*): [M^+^], found 446.31, C_26_H_27_N_3_O_4_ for 445.20.

#### 5‐(1‐(3‐Bromophenyl)‐3‐(1‐ethyl‐1*H*‐indol‐3‐yl)‐3‐oxopropyl)‐1,3‐dimethylpyrimidine‐2,4,6(1*H*,3*H*,5*H*)‐trione (5j)

Yield 187 mg (0.37 mmol, 36.7 %); m.p. 130–131 °C; ^1^H‐NMR (600 MHz, CDCl_3_) *δ*: 1.56 (t, 3H, *J*=5.2 Hz, NCH_2_CH_3_), 3.11 (s, 3H, NCH_3_), 3.16 (s, 3H, NCH_3_), 3.34–3.37 (dd, 1H, *J*=11.2 Hz, 4.0 Hz, CH_2(a)_), 3.92–3.97 (m, 2H CH_2(e)_& CH), 4.24 (q, 2H, *J*=4.8 Hz, NCH_2_CH_3_), 4.41–4.43 (m, 1H, CH), 7.10 (d, 1H, *J*=5.2 Hz, Ar−H), 7.15 (t, 1H, *J*=5.2 Hz, Ar−H), 7.29–7.32 (m, 2H, Ar−H), 7.33 (d, 1H, *J*=1.2 Hz, Ar−H), 7.37–7.39 (m, 2H, Ar−H), 7.97 (s, 1H,Ar−H), 8.37–8.38 (m, 1H, Ar−H); ^13^C‐NMR (150 MHz, CDCl_3_) *δ*: 15.2, 28.1, 28.2, 40.9, 41.9, 44.2, 53.1, 109.8, 116.4, 122.7, 122.8, 123.4, 126.4, 130.2, 130.4, 131.2, 131.7, 134.1, 136.5, 138.2, 141.2, 150.9, 167.7, 168.0, 191.9; IR (KBr, cm^−1^) ν_max_=3453, 3119, 3047, 2975, 1748, 1674, 1633, 1528, 1464, 1426, 1371, 1282, 1204, 1118, 1059, 1001;[Anal. Calcd. for C_25_H_24_BrN_3_O_4_: C, 58.83; H, 4.74; N, 8.23; Found: C, 58.69; H, 4.47; N, 8.45]; LC/MS (ESI, *m/z*): [M^+^], found 510.18, C_25_H_24_BrN_3_O_4_ for 509.10.

#### 5‐(3‐(1‐Ethyl‐1*H*‐indol‐3‐yl)‐3‐oxo‐1‐(4‐(trifluoromethyl)phenyl)propyl)‐1,3‐dimethylpyrimidine‐2,4,6(1*H*,3*H*,5*H*)‐trione (5k)

Yield 198 mg (0.40 mmol, 39.7 %); m.p. 168–169 °C; ^1^H‐NMR (600 MHz, CDCl_3_) *δ*: 1.55 (t, 3H, *J*=5.2 Hz, NCH_2_CH_3_), 3.11 (s, 3H, NCH_3_), 3.17 (s, 3H, NCH_3_), 3.40 & 3.43 (dd, 1H, *J*=11.2 Hz, 4.0 Hz, CH_2(a)_), 3.98 & 4.01 (dd, 1H, *J*=11.2 Hz, 4.0 Hz, CH_2(e)_), 4.03 (d, 1H, *J*=0.8 Hz, CH), 4.24 (q, 2H, *J*=4.8 Hz, NCH_2_CH_3_), 4.55–4.57 (m, 1H, CH), 7.26–7.33 (m, 2H, Ar−H), 7.35–7.38 (m, 3H, Ar−H), 7.54 (d, 2H, *J*=5.6 Hz, Ar−H), 7.97 (s, 1H,Ar−H), 8.33–8.35 (m, 1H, Ar−H); ^13^C‐NMR (150 MHz, CDCl_3_) *δ*: 15.2, 28.6, 28.3, 41.1, 41.8, 43.4, 52.8, 109.8, 116.3, 122.6, 122.8, 123.4, 125.6, 1257, 126.7, 128.2, 134.0, 136.5, 143.7, 150.9, 167.7, 167.8, 192.0; IR (KBr, cm^−1^) ν_max_=3423, 3119, 2982, 1749, 1688, 1636, 1525, 1461, 1421, 1380, 1326, 1207, 1159, 1116, 1070; [Anal. Calcd. for C_26_H_24_F_3_N_3_O_4_: C, 62.52; H, 4.84; N, 8.41; Found: C, 62.33; H, 5.11; N, 8.63]; LC/MS (ESI, *m/z*): [M^+^], found 500.20, C_26_H_24_F_3_N_3_O_4_ for 499.17.

#### 5‐(3‐(1‐Ethyl‐1*H*‐indol‐3‐yl)‐3‐oxo‐1‐(thiophen‐2‐yl)propyl)‐1,3‐dimethylpyrimidine‐2,4,6(1*H*,3*H*,5*H*)‐trione (5l)

Yield 224 mg (0.54 mmol, 53.7 %); m.p. 154–155 °C; ^1^H‐NMR (600 MHz, CDCl_3_) *δ*: 1.56 (t, 3H, *J*=4.8 Hz, NCH_2_CH_3_), 3.15 (s, 3H, NCH_3_), 3.17 (s, 3H, NCH_3_), 3.39–3.43 (m, 2H, CH_2(a)_), 3.98–4.01 (dd, 1H, *J*=11.2 Hz, 6.4 Hz, CH_2(e)_), 4.05 (d, 1H, *J*=2.4 Hz, CH), 4.24 (q, 2H, *J*=4.8 Hz, NCH_2_CH_3_), 4.82–4.83 (m, 1H, CH), 6.86 (d, 1H, *J*=2.0 Hz, Ar−H), 6.90 (t, 1H, *J*=2.8 Hz, Ar−H), 7.16 (d, 1H, *J*=3.6 Hz, Ar−H), 7.29–7.37 (m, 2H, Ar−H), 7.37–7.38 (m, 1H, Ar−H), 7.96 (s, 1H,Ar−H), 8.38–8.40 (m, 1H, Ar−H);^13^C‐NMR (150 MHz, CDCl_3_) *δ*: 15.2, 28.2, 28.4, 39.6, 41.8, 42.7, 53.0, 109.7, 116.4, 122.7, 123.3, 124.9, 125.7, 126.4, 126.9, 128.3, 134.2, 136.5, 141.5, 151.2, 167.6, 167.9, 191.9; IR (KBr, cm^−1^) ν_max_=3458, 3104, 3051, 2974, 2928, 1748, 1662, 1563, 1530, 1460, 1425, 1381, 1317, 1273, 1200, 1148, 1128, 1051; [Anal. Calcd. for C_23_H_23_N_3_O_4_S: C, 63.14; H, 5.30; N, 9.60; Found: C, 63.35; H, 5.41; N, 9.48]; LC/MS (ESI, *m/z*): [M^+^], found 438.10, C_23_H_23_N_3_O_4_S for 437.14.

#### 5‐(3‐(1‐Ethyl‐1*H*‐indol‐3‐yl)‐1‐(furan‐2‐yl)‐3‐oxopropyl)‐1,3‐dimethylpyrimidine‐2,4,6(1*H*,3*H*,5*H*)‐trione (5m)

Yield 230 mg (0.55 mmol, 54.6 %); m.p. 188–189 °C; ^1^H‐NMR (600 MHz, CDCl_3_) *δ*: 1.55 (t, 3H, *J*=4.8 Hz, NCH_2_CH_3_), 3.18 (s, 3H, NCH_3_), 3.21 (s, 3H, NCH_3_), 3.39–3.41 (dd, 1H, *J*=11.2 Hz, 6.4 Hz, CH_2(a)_), 3.81 & 3.84 (dd, 1H, *J*=11.2 Hz, 6.0 Hz, CH_2(e)_), 3.96 (d, 1H, *J*=2.4 Hz, CH), 4.24 (q, 2H, *J*=4.8 Hz, NCH_2_CH_3_), 4.57–4.59 (m, 1H, CH), 6.11 (d, 1H, *J*=2.4 Hz, Ar−H), 6.26 (q, 1H, *J*=1.6 Hz, Ar−H), 7.26–7.37 (m, 1H, Ar−H), 7.29–7.32 (m, 2H, Arq‐H), 7.37–7.38 (m, 1H, Ar−H), 7.95 (s, 1H,Ar−H), 8.38–8.40 (m, 1H, Ar−H); ^13^C‐NMR (150 MHz, CDCl_3_) *δ*: 15.2, 28.3, 28.4, 38.1, 39.7, 41.8, 51.5, 107.0, 109.7, 110.5, 116.4, 122.7, 123.4, 126.5, 129.9, 134.2, 136.5, 142.2, 151.4, 152.8, 167.6, 167.9, 191.8; IR (KBr, cm^−1^) ν_max_=3427, 3116, 2978, 2932, 1744, 1642, 1655, 1530, 1460, 1422, 1394, 1272, 1202, 1145, 1114; [Anal. Calcd. for C_23_H_23_N_3_O_5_: C, 65.55; H, 5.50; N, 9.97; Found: C, 65.73; H, 5.63; N, 10.09]; LC/MS (ESI, *m/z*): [M^+^], found 422.20, C_23_H_23_N_3_O_5_ for 421.16.

#### 5‐(3‐(1‐Ethyl‐1*H*‐indol‐3‐yl)‐3‐oxo‐1‐(3,4,5‐trimethoxyphenyl)propyl)‐1,3‐dimethylpyrimidine‐2,4,6(1*H*,3*H*,5*H*)‐trione (5n)

Yield 185 mg (0.35 mmol, 35.5 %); m.p. 170–171 °C; ^1^H‐NMR (600 MHz, CDl_3_) *δ*: 1.56 (t, 3H, *J*=4.8 Hz, NCH_2_CH_3_), 3.12 (s, 3H, NCH_3_), 3.17 (s, 3H, NCH_3_), 3.34–3.36 (dd, 1H, *J*=10.8 Hz, 4.0 Hz, CH_2(a)_), 3.80 (s, 3H, OCH_3_), 3.81 (s, 3H, OCH_3_), 3.82 (s, 3H, OCH_3_), 3.95–3.99 (m, 2H, CH_2(e)_ & CH), 4.24 (q, 2H, *J*=4.8 Hz, NCH_2_CH_3_), 4.39–4.41 (m, 1H, CH), 6.39 (s, 2H,Ar−H), 7.30–7.33 (m, 2H, Ar−H), 7.37–7.39 (m, 1H, Ar−H), 7.97 (s, 1H,Ar−H), 8.38–8.40 (m, 1H, Ar−H); ^13^C‐NMR (150 MHz, CDCl_3_) *δ*: 15.2, 28.2, 28.3, 41.4, 41.9, 44.7, 51.1, 53.0, 60.8, 60.9, 109.7, 115.1, 116.5, 122.7, 122.8, 123.3, 126.5, 132.6, 134.0, 134.7, 136.5, 151.0, 153.2, 168.1, 168.3, 192.4; IR (KBr, cm^−1^) ν_max_=3435, 3105, 2983, 2946, 2839, 1748, 1677, 1646, 1589, 1526, 1512, 1423, 1376, 1331, 1243, 1209, 1127, 1104, 1003; [Anal. Calcd. for C_28_H_31_N_3_O_7_: C, 64.48; H, 5.99; N, 8.06; Found: C, 64.31; H, 6.09; N, 8.24]; LC/MS (ESI, *m/z*): [M^+^], found 522.20, C_28_H_31_N_3_O_7_ for 521.22.

#### 5‐(3‐(1‐Ethyl‐1H‐indol‐3‐yl)‐1‐(naphthalen‐2‐yl)‐3‐oxopropyl)‐1,3‐dimethylpyrimidine‐2,4,6(1H,3H,5H)‐trione (5o)

Yield 178 mg (0.37 mmol, 37.0 %); m.p. 85–86 °C; ^1^H‐NMR (600 MHz, CDCl_3_) *δ*: 1.56 (t, 3H, *J*=4.8 Hz, NCH_2_CH_3_), 3.02 (s, 3H, NCH_3_), 3.09 (s, 3H, NCH_3_), 3.45–3.47 (dd, 1H, *J*=10.8 Hz, 3.6 Hz, CH_2(a)_), 4.08–4.15 (m, 2H, CH_2(e)_ & CH), 4.24 (q, 2H, *J*=4.8 Hz, NCH_2_CH_3_), 4.60–4.64 (m, 1H, CH), 7.29–7.32 (m, 3H, Ar−H), 7.36–7.39 (m, 1H, Ar−H), 7.45–7.47 (m, 2H, Ar−H), 7.63 (s, 1H,Ar−H), 7.75–7.79 (m, 3H, Ar−H), 8.00 (s, 1H,Ar−H), 8.39–8.40 (m, 1H, Ar−H); ^13^C‐NMR (150 MHz, CDCl_3_) *δ*: 15.4, 28.4, 28.8, 41.2, 41.8, 44.2, 52.4, 108.9, 113.5, 116.5, 121.5, 121.9, 123.5, 125.3, 126.0, 126.2, 126.5, 127.1, 127.6, 128.2, 128.9, 130.7, 134.3, 137.2, 140.6, 158.2, 168.6, 169.4, 192.4; IR (KBr, cm^−1^) ν_max_=3438, 3106, 2983, 2944, 2832, 1745, 1678, 1643, 1585, 1528, 1516, 1424, 1370, 1332, 1243, 1205, 1128, 1103, 1005; [Anal. Calcd. for C_29_H_27_N_3_O_4_: C, 72.33; H, 5.65; N, 8.73; Found: C, 72.49; H, 5.41; N, 8.85]; LC/MS (ESI, *m/z*): [M^+^], found 482.30, C_29_H_27_N_3_O_4_ for 481.20.

#### 5‐(3‐(1‐Ethyl‐1*H*‐indol‐3‐yl)‐1‐(4‐nitrophenyl)‐3‐oxopropyl)‐1,3‐ dimethylpyrimidine‐2,4,6(1*H*,3*H*,5*H*)‐trione (5q)

Yield 165 mg (0.35 mmol, 34.6 %); m.p. 198–199 °C; ^1^H‐NMR (600 MHz, CDCl_3_) *δ*: 1.55 (t, 3H, *J*=4.8 Hz, NCH_2_CH_3_), 2.21 (s, 3H, CH_3_), 3.23 (s, 3H, NCH_3_), 3.34–3.37 (dd, 1H, *J*=12.0 Hz, 4.4 Hz, CH_2(a)_), 3.93 & 3.96 (dd, 1H, *J*=12.0 Hz, 4.4 Hz, CH_2(e)_), 4.21–4.26 (m, 4H, CH & NCH_2_CH_3_), 7.28–7.31 (m, 2H, Ar−H), 7.38 (t, 3H, *J*=6.0 Hz, Ar−H), 7.88 (s, 1H,Ar−H), 8.17 (d, 2H, *J*=6.0 Hz, Ar−H), 8.25–8.27 (m, 1H, Ar−H); ^13^C‐NMR (150 MHz, CDCl_3_) *δ*: 15.2, 29.0, 29.3, 38.7, 41.9, 42.5, 50.2, 109.9, 116.2, 122.4, 122.9, 123.8, 125.9, 126.5, 127.1, 129.3, 133.6, 142.7, 144.8, 151.5, 167.8, 168.6, 192.1; IR (KBr, cm^−1^) ν_max_=3379, 3125, 2980, 1761, 1690, 1634, 1532, 1517, 1460, 1381, 1215, 1078; [Anal. Calcd. for C_25_H_24_N_4_O_6_: C, 63.02; H, 5.08; N, 11.76; Found: C, 62.84; H, 5.25; N, 12.01]; LC/MS (ESI, *m/z*): [M^+^], found 477.20, C_25_H_24_N_4_O_6_ for 476.17

### Single Crystal X‐ray Crystallography

The compound of **5g** was obtained as single crystals by slow evaporation from ethanol solution of the pure compound at room temperature. Data were collected on a Bruker APEX‐II D8 Venture area diffractometer, equipped with graphite monochromatic Mo *K*α radiation, λ=0.71073 Å at 293 (2) K. Cell refinement and data reduction were carried out by Bruker SAINT. SHELXT^15^ was used to solve structure. The final refinement was carried out by full‐matrix least‐squares techniques with anisotropic thermal data for non‐hydrogen atoms on. CCDC 1877313 contains the supplementary crystallographic data for this compound can be obtained free of charge from the Cambridge Crystallographic Data Centre *via*www.ccdc.cam.ac.uk/data_request/cif.

### Biological Activitiy Assays

#### Reagents

α‐glucosidase type 1 from baker‘s yeast (G5003; Sigma‐Aldrich, St. Louis, MO, USA), *p*‐nitrophenyl α‐D‐glucopyranoside (N1377, Sigma‐Aldrich), sodiumphosphatemonobasic (S3139, Sigma‐Aldrich), sodiumphosphatedibasic (S5136, Sigma‐Aldrich), andacarbose (A8980, Sigma‐Aldrich), DMSO (Dimethylsulfoxide), α‐amylase from *Aspergillus oryzae* (Sigma Aldrich), starch, DNS (3, 5‐dinitrosalicylic acid), sodiumpotassiumtartratetetrahydrate.

#### α‐Glucosidase Inhibition Assay

*Concentration of α‐glucosidase and substrate*. Sodium phosphate buffer (0.1 M) was adjusted by 0.1 N HCl to pH 7.0 with a pH meter (Thermo Fisher Scientific Inc., Waltham, MA, USA). *p*‐Nitrophenyl α‐D‐glucopyranoside (10 mM) and α‐glucosidase solutions (1 U/ml) were solubilized in 0.1 M sodium phosphate buffer (pH 7.0). All the reagents were manufactured shortly before use and warmed to 37 °C in a water bath. Sodium phosphate buffer (0.1 M, 158 *μ*l per well) was added to a 96‐well plate. α‐Glucosidase (20 *μ*l) and 2 *μ*l of sample were added to 20 *μ*l of *p*‐nitrophenyl α‐D‐glucopyranoside. In the 200‐*μ*l final reaction volume (0.02 U/well, 0.1 U/ml) the substrate concentration was adjusted to 10 mM. The background signal due to the sample color was measured at 405 nm with the PerkinElmer Wallac Victor3 spectrophotometer (PerkinElmer, Waltham, MA, USA) prior to adding the enzyme. Immediately following α‐glucosidase addition, absorbance was measured at 405 nm 8 times at 1‐min intervals.^16^

#### α Amylase Assay

Briefly, 250 *μ*L (0.4 mg/mL) was preincubated with 250 *μ*L of α‐amylase solution for 10 min at 25 °C in one set of tubes. In another set of tubes α‐amylase was preincubated with 250 *μ*L of phosphate buffer (pH 6.9). 250 μL of starch solution at increasing concentrations (0.2–1 % (w/v)) was added to both sets of reaction mixtures to start the reaction. The mixture was then incubated for 10 min at 25 °C and then boiled for 15 min after the addition of 250 *μ*L of DNS to stop the reaction. The amount of reducing sugars released was determined spectrophotometrically using a maltose standard curve and converted to reaction velocities.

##### Calculation of Inhibition Efficiency

The inhibitory concentration 50 % (IC_50_) values were determined from the plots of percent inhibition versus log inhibitor concentration and calculated by logarithmic regression analysis from the mean inhibitory values.

#### Docking Studies

A virtual library of designed compounds was energy minimized using the MMFF94 force field, which was followed by the generation of multi‐conformers using the Omega application. The entire energy‐minimized library was docked with the prepared catalytic domain of (PDB code: 4UAC)^12^ using the FRED application in OpenEye software13b to generate a physical property (ΔG) reflecting the predicted energy profile of the ligand‐receptor complex. The Vida application can be employed as a visualization tool to show the potential binding interactions of the ligands with the receptor of interest.

## 3 Conclusion

The present study mainly focuses on the synthesis of novel indole‐pyrimidine based chemical entities for the improved anti‐diabetic activity. The new series of indole‐ pyrimidine based compounds obtained via bimetallic catalytic system which has a dramatic effect in promoting the Michael addition reaction. The synthesized compounds screened against wide range of α‐glucosidase inhibition and α ‐amylase assay inhibitory activity. Docking study describes that both barbiturate and acyl indole parts participate in HB while the aryl linker occupy the receptor through lipophilic‐lipophilic interactions.

## Conflict of interest

The authors report no declarations of interest.
